# Low Ankle‐Brachial Index Is Associated With Albuminuria and Diabetic Kidney Disease in Type 2 Diabetes; A Cross Sectional Study

**DOI:** 10.1002/edm2.70115

**Published:** 2025-11-07

**Authors:** Maryam Hedayati Moshkele, Saeed Mirmoosavi, Mohammad Taghi Najafi, Sahar Karimpour, Alireza Esteghamati, Manouchehr Nakhjavani, Soghra Rabizadeh

**Affiliations:** ^1^ Endocrinology and Metabolism Research Center (EMRC), Vali‐Asr Hospital Tehran University of Medical Sciences Tehran Iran; ^2^ Urology Research Center Tehran University of Medical Sciences Tehran Iran; ^3^ Nephrology Research Center Tehran University of Medical Sciences Tehran Iran

**Keywords:** diabetic kidney disease, peripheral artery disease, type 2 diabetes, vascular complications

## Abstract

**Background:**

Micro‐ and macrovascular complications of type 2 diabetes, including diabetic kidney disease (DKD) and peripheral artery disease (PAD), impose a significant burden on patients and healthcare systems. Diabetes is associated with a twofold higher risk of PAD. No studies from the Middle East have examined the relationship between PAD and DKD. Given the shared vascular pathology of DKD and PAD, this study investigated the association between a low ankle‐brachial index (ABI) and albuminuria/DKD.

**Method:**

This analytical cross‐sectional study included patients diagnosed with diabetes per ADA criteria. ABI was measured using a four‐channel automated oscillometric sphygmomanometer after 30 min of rest. DKD was defined as albuminuria [urinary albumin > 30 mg/g cr] and/or eGFR < 60 mL/min/1.73 m^2^. Binary logistic regression assessed the association between ABI and both albuminuria and DKD, adjusting for confounders.

**Results:**

Among 151 patients, 48% had PAD in at least one limb defined by ABI ≤ 0.9. ABI ≤ 0.9 was associated with albuminuria (OR = 2.32, 95% CI: 1.15–4.7, *p* = 0.01), which remained significant after adjustment for age, sex, hypertension, and coronary artery disease (CAD) (OR = 2.97, 95% CI: 1.28–6.8, *p* = 0.02). Similarly, ABI ≤ 0.9 increased the odds of DKD (OR = 2.7, 95% CI: 1.39–5.2, *p* = 0.003), and after adjustment, the association remained significant (OR = 2.99, 95% CI: 1.05–8.4, *p* = 0.04). ABI < 0.8 further increased the risk of DKD (OR = 7.5, 95% CI: 1.9–29, *p* = 0.003).

**Conclusion:**

A reduced ABI (< 0.9) is independently associated with both albuminuria and DKD in type 2 diabetes, with a stronger association at ABI < 0.8. These findings highlight the potential role of ABI as a simple, non‐invasive, and accessible screening tool for identifying patients at increased risk of DKD.

## Graphical abstract

Low ankle brachial index (< 0.9) in patients with type 2 diabetes is associated with 3‐fold higher odds of both albuminuria and diabetic kidney disease.

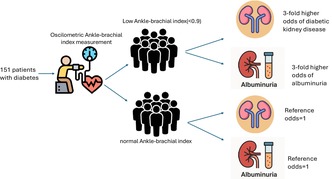

## 1 Introduction

Diabetes mellitus is a long‐term condition characterised by persistent hyperglycemia. It negatively affects the quality of life of patients and is responsible for significant mortality and morbidity [1]. Micro and macrovascular complications of the disease, such as diabetic nephropathy, retinopathy, neuropathy, and atherosclerosis, put a huge burden on patients and healthcare systems [2]. There are multiple mechanisms by which chronic hyperglycemia leads to these vascular conditions. Hyperglycemia promotes the formation of Advanced Glycation End products (AGEs), oxidative stress, and a proinflammatory microenvironment, contributing to diabetes‐related complications [3].

Diabetic kidney disease (DKD), a form of chronic kidney disease caused by diabetes, is the leading cause of end‐stage renal disease. This microvascular complication usually begins with microalbuminuria in the early stages and, if it is not controlled, can progress to overt proteinuria and a decline in the glomerular filtration rate (GFR) [4]. Since reversing microvascular changes is not yet feasible, the most important treatment for DKD is prevention [5].

Peripheral artery disease (PAD), marked by reduced blood flow due to limb vessel occlusion, commonly results from atherosclerosis [6]. Diabetes mellitus (DM) is a major risk factor for PAD leading to at least a twofold increased risk of developing PAD [7]. PAD is often a peripheral manifestation of coronary and cerebrovascular atherosclerosis, which increases the risk of cardiovascular events. Ankle brachial index (ABI)—the ratio of systolic blood pressure at the ankle to that in the brachial artery—is a non‐invasive index used to diagnose PAD with high accuracy [8]. According to the American Heart Association, normal values are between 1 and 1.4 [9].

As both DKD and PAD are vascular complications of diabetes, some studies have investigated the inter‐relationship between the two conditions. They have shown that PAD is a risk factor for increased mortality in patients with DKD [10]. Diabetic nephropathy has also been linked to an increased risk of developing PAD [11].

Both DKD and PAD share common pathophysiological mechanisms rooted in chronic hyperglycemia. Long‐standing diabetes induces endothelial dysfunction through oxidative stress, reduced nitric oxide bioavailability, and the accumulation of advanced glycation end‐products (AGEs), all of which impair vascular homeostasis and contribute to a pro‐atherogenic environment [1, 3]. Endothelial dysfunction not only initiates atherosclerosis in large arteries, manifesting as PAD, but also disrupts glomerular filtration and capillary integrity, promoting DKD. Furthermore, arterial stiffness has been increasingly recognised as a shared mechanism linking macrovascular and microvascular complications of diabetes. Increased arterial stiffness raises systolic pressure and pulsatile load on small vessels, accelerating kidney damage while also compromising peripheral circulation [12, 13]. These interrelated processes highlight a unifying vascular pathology underlying both PAD and DKD, which may explain the observed clinical association between the two conditions.

Despite the research cited above, there are limited studies in the literature on the correlation between PAD and DKD in type 2 diabetes with limited studies in the Middle East [11, 14]. This study aimed to investigate whether low ankle‐brachial index (ABI) at different thresholds (such as < 0.9 and < 0.8) is associated with albuminuria and DKD.

## 2 Materials and Methods

The study was an analytical cross‐sectional study. Patients were recruited from a tertiary center affiliated with Tehran University of Medical Sciences between March 2023 and May 2024. The inclusion criteria were adults with type 2 diabetes mellitus. Exclusion criteria were: (1) patients with malignancy, (2) pregnancy, (3) type 1 diabetes mellitus, (4) end‐stage renal disease defined as GFR < 15 mL/min/1.73 calculated by the Modification of Diet in Renal Disease formula (MDRD) equation, (5) patients undergoing haemodialysis and (6) blood transfusion within the last three months. A sample size calculation was carried out using G*Power software, version 3.1.9.7. Based on a previous study by Dong et al. [15] the odds of low ABI and diabetic nephropathy (DN) were 2.2 (1.18–4.07 95% CI). A total of 146 patients were required assuming an *α* error = 0.05 and a power of 95%. The formula by which sample size was calculated is as follows:

**Table:** 

*z* tests—Logistic regression	
Options:	Large sample *z*‐test, Demidenko (2007) with var corr
Analysis:	A priori: Compute required sample size
Tail(s)	Two
Input:	Odds ratio = 2.2
	Pr(*Y* = 1|*X* = 1) H0 = 0.19
	*α* err prob = 0.05
	Power (1 − *β* err prob) = 0.95
	*R* ^2^ other *X* = 0
	*X* distribution = Normal
	*X* parm *μ* = 0
	*X* parm *σ* = 1
Output:	Critical *z* = 1.9599640
	Total sample size = 146
	Actual power = 0.9505217

Diagnosis of diabetes was made according to the American Diabetes Association (ADA) criteria [16]. Patients with albuminuria were selected based on multiple urinary samples with consistent albuminuria. Patients with a single episode of albuminuria with subsequent normoalbuminuria were excluded. Estimated glomerular filtration rate (eGFR) was calculated using the Modification of Diet in Renal Disease formula (MDRD) [17]. Albuminuria was defined as micro/macroalbuminuria (urinary albumin > 30 mg/g cr) in a 24 h urinary specimen in at least two separate measurements. DKD was defined as either albuminuria (urinary albumin > 30 mg/g cr/24 h) and/or a declined GFR (< 60 mL/min/1.73 m^2^). This study was in accordance with the Helsinki Declaration and was approved by the local ethics committee under the code IR.TUMS.IKHC.REC.1402.256. Informed consent for publication and participation was acquired from all patients involved in the study.

### 2.1 ABI Measurement

ABI was measured using an SOT device, a four‐channel automated oscillometric sphygmomanometer made in Austria, after 30 min of rest. The SOT device used for ABI measurement was certified according to ISO 13485:2016 (Registration Number: SI—M‐166). BP was measured with the patient in a supine position. An experienced operator placed cuffs on both ankles and arms. The device automatically calculated the ABI for both the right and left sides. Both left and right ABI as well as total ABI were studied. Total ABI ≤ 0.9 was defined as either left or right ABI ≤ 0.9. The same was applied for ABI ≤ 0.8. PAD was defined as total ABI ≤ 0.9. ABI measurement using a validated four‐channel automated oscillometric sphygmomanometer has been shown to offer acceptable accuracy and ease of use for clinical and research settings, particularly where Doppler ultrasound is not feasible due to time or resource constraints [18].

### 2.2 Physical and Laboratory Measurements

Baseline data such as age, sex, duration of diabetes, history of comorbidities, height, and weight were collected on the same day of ABI measurement. Venous blood samples were drawn after 12 h of fasting. Fasting plasma glucose (FPG) was assessed using the glucose oxidase method, while glycated haemoglobin A1c (HbA1c) was measured via high‐performance liquid chromatography (HPLC) (DS5, DREW, England). Cholesterol, triglycerides (TG), low‐density lipoprotein cholesterol (LDL‐C), high‐density lipoprotein cholesterol (HDL‐C), as well as urea, creatinine, and uric acid levels were determined using enzymatic techniques (Parsazmun; Auto Analyser, BT‐3000(plus), Biotechnica).

### 2.3 Statistical Analysis

Quantitative and qualitative variables were expressed as mean, standard deviation (SD), and percentage, respectively. Median and IQR were reported where the distribution was not normal. Independent *t*‐test and Mann–Whitney *U* test were used for comparing continuous variables between two groups. Chi‐square test was run for categorical variables. Univariate and multivariate binary logistic regression were used to assess the association of ABI and both albuminuria and DKD while adjusting for confounding variables.

All statistical analyses were conducted by SPSS package version 24.0 for Windows (IBM Corporation, New York, USA). A *p*‐value of lower than 0.05 was considered statistically significant for all tests. The nomogram was drawn by Python version 3.8.

## 3 Results

A total of 151 patients with type 2 diabetes with and without albuminuria were studied. The mean age of participants was 59.2 ± 10.1, and 45% of them were male. Forty‐eight patients (32%) had albuminuria, and 31 patients (20.5%) had chronic kidney disease (GFR < 60 mL/min/1.73 m^2^). Sixty‐eight patients (45%) had DKD defined as either persistent albuminuria or reduced GFR. Baseline and demographic characteristics of patients based on the presence of albuminuria are presented in Table 1. Patients with albuminuria were significantly older (*p* = 0.047). Both groups were sex matched. Other baseline characteristics were not significantly different between the two groups.

**TABLE 1:** Baseline characteristics of patients with and without albuminuria.

Characteristic	Group			
	Total (151)	Without albuminuria (103)	With albuminuria (48)	*p*
Age	59.2 ± 10.1	58.8 ± 10.4	61.6 ± 9.1	0.047
Sex (male)	67 (44%)	47 (46%)	20 (42%)	0.64
BMI	29.4 ± 4.8	29.8 ± 5.1	28.7 ± 4.2	0.26
Duration of diabetes (years)	10 (10)^a^	10 (9.5)^a^	13 (12.3)^a^	0.22
Waist circumference (cm)	101.2 ± 15.1	102 ± 11.9	99.4 ± 20.8	0.38
Albuminuria (mg/24 h)	58.8 ± 143.5	13.2 ± 8.2	157 ± 226.3	< 0.001
HTN	52.7%	52%	53%	0.89
History of CAD	20%	20.9%	18%	0.71
Diabetic foot ulcer	9.9%	9.7%	10.4%	0.89
Right ABI ≤ 0.9	32%	26%	46%	0.02
Left ABI ≤ 0.9	40%	33%	56%	< 0.01
GFR	79.9 ± 23.3	82 ± 22.7	75.4 ± 24	0.11
GFR < 60 mL/min/1.73^2^	20.5%	19.4%	22.9%	0.62
Triglyceride	136 (92)^a^	130 (77)^a^	166 (106)^a^	0.06
Cholesterol	155.1 ± 57	153.6 ± 42	158.4 ± 80.2	0.34
LDL	77.5 ± 36.1	81 ± 36.5	70 ± 34.5	0.11
HDL	42.2 ± 11.8	42 ± 12.5	43.3 ± 10.4	0.46
AST	23 ± 24.8	21.1 ± 11.01	26 ± 40.6	0.28
ALT	25.5 ± 25.3	25.2 ± 19	26 ± 35.4	0.88
FBS	197.4 ± 97	195.8 ± 99.1	201 ± 94	0.76
HbA1c	9.2 ± 2.7	9 ± 2.5	9.5 ± 3.1	0.24
Uric acid	4.8 ± 2.1	4.6 ± 2.4	5.1 ± 1.3	0.34

^a^ Non‐normally distributed data are presented as median (IQR).

### 3.1 Prevalence of PAD According to Ankle Brachial Index

**FIGURE 1:**
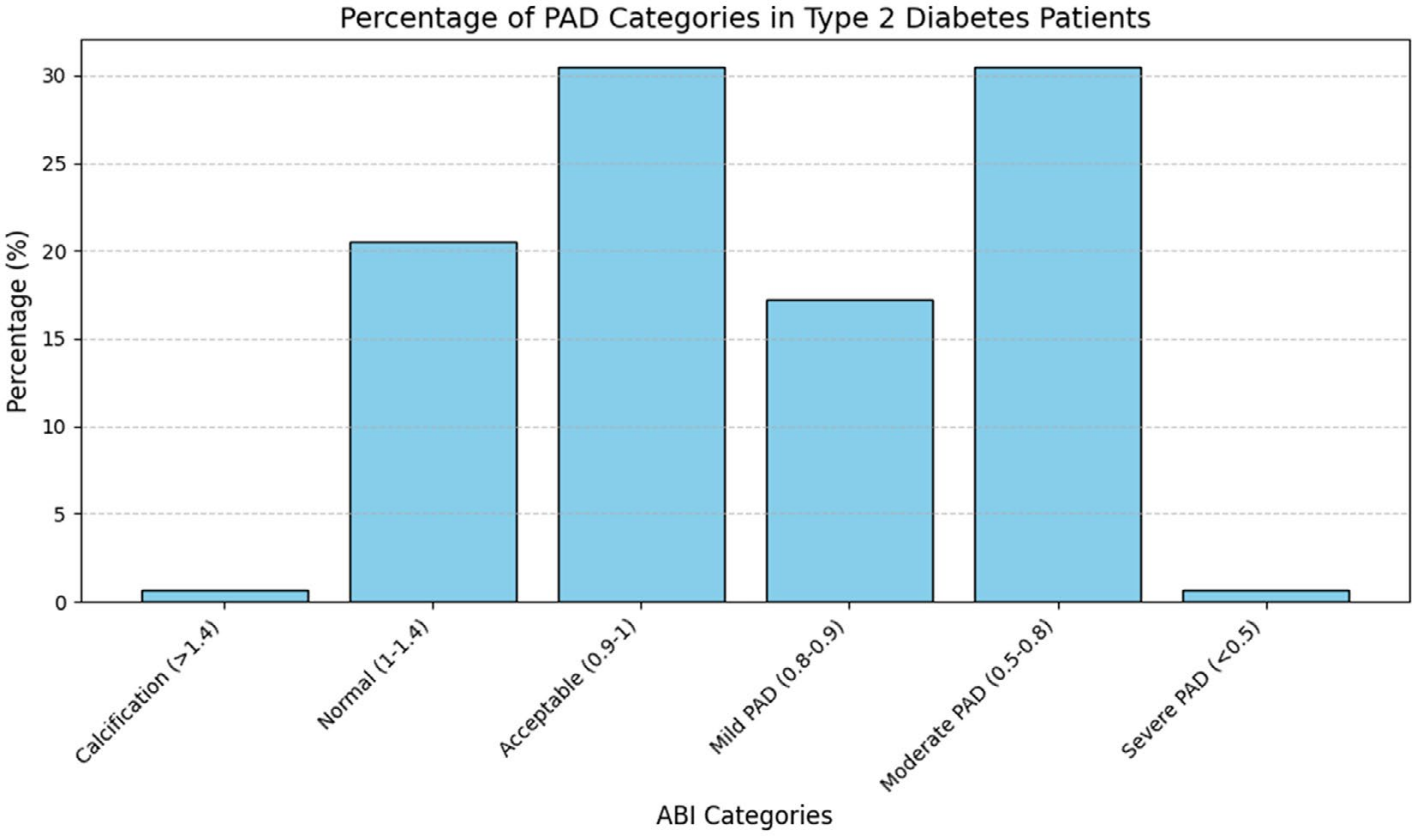
Frequency and severity of PAD in patients with type 2 diabetes.

The prevalence of PAD varied based on the side of the ABI measurement. Forty‐nine patients (32.5%) had a right ABI ≤ 0.9, and 61 patients (40%) had a left ABI ≤ 0.9. Overall, 48% of patients had PAD in at least one side. We further categorised patients into six groups based on the value of ABI as shown in Figure 1. Normal ABI was defined as 1 < ABI < 1.4, and 0.9 < ABI ≤ 1 was acceptable. Values higher than 1.4 showed calcification. 0.8 < ABI ≤ 0.9 was labelled as mild PAD, and 0.5 < ABI ≤ 0.8 and ABI ≤ 0.5 respectively showed moderate and severe PAD. The two largest groups were those with moderate PAD (ABI = 0.5–0.8) and those with near‐normal ABI values (0.9 < ABI ≤ 1), each comprising 30.5% of patients. There was no significant difference in the prevalence of ABI ≤ 0.9 between males and females (53% vs. 44%, *p* = 0.23). Also, the mean age of patients with or without PAD was not significantly different (58.1 ± 8.9 vs. 60.2 ± 11.2, *p* = 0.2).

### 3.2 Association of ABI With Albuminuria and Diabetic Kidney Disease

In the unadjusted model, ABI ≤ 0.9 had an odds ratio of 2.32 for albuminuria (*p* = 0.01). The odds ratio remained significant after adjustment for age, sex, hypertension, and history of coronary artery disease (CAD) (OR = 2.97, *p* = 0.02). Right and left ABI ≤ 0.9 were also significant risk factors for albuminuria after adjustment for the above‐mentioned covariates (r‐ABI < 0.9, OR = 2.66, *p* = 0.017; l‐ABI < 0.9, OR = 2.82, *p* = 0.013). The above‐mentioned models are shown in Table 2.

**TABLE 2:** Association of ABI < 0.9 with albuminuria using univariate and multivariate logistic models.

	OR	95% CI	*p*
Univariate			
rABI < 0.9	2.38	1.16–4.8	0.018
lABI < 0.9	2.6	1.3–5.2	0.007
Total ABI < 0.9^a^	2.32	1.15–4.7	0.02
Multivariate^b^			
rABI < 0.9	2.66	1.19–5.9	0.017
lABI < 0.9	2.82	1.24–6.4	0.013
Total ABI < 0.9	2.97	1.28–6.8	0.011

^a^ Total ABI < 0.9 means at least one side has ABI < 0.9.

^b^ Adjusted for age, sex, hypertension, and history of CAD.

**FIGURE 2:**
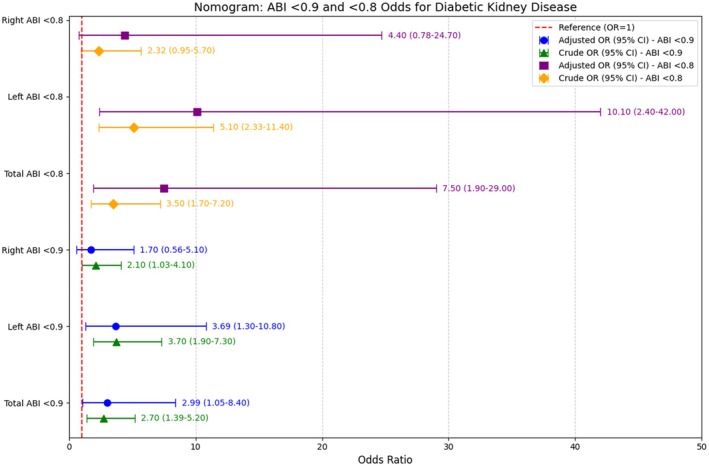
Odds ratios for the association of ABI < 0.9 and < 0.8 with diabetic kidney disease in patients with type 2 diabetes, adjusted for confounders.

Univariate logistic regression depicted that ABI ≤ 0.9 had an odds ratio of 2.7 for DKD. After adjustment for potential confounders, the odds ratio of ABI ≤ 0.9 for having DKD was 2.99 with a 95% CI of 1.05–8.4, *p* = 0.04. (Table 3 and Figure 2) Both right and left ABI < 0.9 were also significantly associated with DKD.

**TABLE 3:** Odds ratio of ABI < 0.9 and < 0.8 for diabetic kidney disease.

	Odds ratio	95% CI	*p*
Univariate			
Total ABI < 0.9^a^	2.7	1.39–5.2	0.003
Total ABI < 0.8	3.5	1.7–7.2	< 0.001
Multivariate^b^			
Total ABI < 0.9	2.99	1.05–8.4	0.04
Total ABI < 0.8	7.5	1.9–29	0.003

^a^ Total ABI < 0.9 means at least one side has ABI < 0.9.

^b^ Adjusted for age, sex, HbA1c, LDL, BMI, duration of diabetes, hypertension, and history of CAD.

We also assessed the association of ABI < 0.8 with DKD. Table 3 shows the odds ratio of ABI < 0.8 for DKD. In the adjusted model, ABI < 0.8 had an odds ratio of 7.5 for DKD (*p* = 0.003).

## 4 Discussion

In this study, we aimed to investigate the association of PAD with albuminuria and DKD in patients with type 2 diabetes. The results demonstrated that a low ABI was independently associated with increased odds of both albuminuria and DKD. ABI < 0.9 raised the likelihood of both albuminuria and DKD about threefold, and ABI < 0.8 was associated with 7.5‐fold higher odds of having DKD.

In this study, almost half of the patients were found to have an ABI < 0.9, indicating a notably high prevalence of PAD in Iranian patients with type 2 diabetes. The prevalence of PAD in patients with type 2 diabetes varies significantly among different studies. For instance, a German study found that 26% of patients with diabetes had PAD based on low ABI measurements [19]. According to a review study, the prevalence of PAD in diabetes ranges from 9% to 55% and it is more prevalent in hospital‐based studies compared to other ones [6, 20]. Longer duration of diabetes has been associated with a higher rate of PAD in previous studies [14, 21]. The relatively high prevalence observed in our study may be attributable to the longer duration of diabetes among participants and the fact that our participants were enrolled from a tertiary center. The gold standard diagnostic test for PAD is CT angiography [22]. However, ABI measurement is a widely accepted non‐invasive alternative with high diagnostic accuracy for PAD. In the absence of advanced imaging modalities, ABI is a feasible, inexpensive option for screening PAD in resource‐limited settings. Previous studies have documented a strong correlation between PAD and both microvascular and macrovascular complications in patients with type 2 diabetes. Cardoso et al. showed that an ABI < 0.9 was associated with a 2.1‐ and 2.7‐fold greater risk of all‐cause and cardiovascular mortality, respectively, in patients with type 2 diabetes [23].

Our finding that ABI < 0.8 was associated with a markedly higher odds of DKD (OR = 7.5, 95% CI: 1.9–29) compared to ABI ≤ 0.9 (OR = 2.99) suggests a dose–response relationship between the severity of PAD and the risk of DKD. ABI values below 0.8 are typically considered indicative of moderate to severe arterial obstruction, implying more advanced atherosclerosis. This stronger association at lower ABI thresholds may reflect a higher burden of systemic vascular dysfunction and impaired renal perfusion, both of which are critical contributors to the progression of DKD. In clinical terms, ABI < 0.8 could serve not only as a marker of PAD but also as an indicator of more advanced or progressive kidney disease in patients with type 2 diabetes. Identifying such patients early may allow for intensified monitoring and therapeutic strategies to slow renal deterioration.

DKD is a complex condition that usually starts with microalbuminuria and, if left untreated, can progress to reduced GFR and finally end‐stage renal disease. Recent studies have also recognised a non‐albuminuric pathway of DKD progression, where patients experience a decline in GFR without prior albuminuria. This phenotype suggests an alternative pathophysiology involving predominantly tubulointerstitial or vascular damage rather than glomerular injury [24, 25]. Studies have shown that patients with non‐albuminuric DKD may present with more pronounced vascular stiffness and higher rates of cardiovascular morbidity, despite the absence of elevated urinary albumin excretion [24, 26]. Nonalbuminuric renal dysfunction may represent a form of DKD that remains undetected due to the early and effective application of anti‐albuminuric treatments—such as renin–angiotensin system inhibitors—and rigorous management of blood glucose, lipids, and blood pressure, either during the initial stages of type 2 diabetes or long before its formal diagnosis [27]. Despite glycemic control and hypertension management, DKD continues to dampen renal function in some patients, suggesting that additional risk factors may contribute to its pathogenesis. Roy et al. identified factors such as older age and female sex as significant risk factors for DKD, whereas moderate alcohol consumption appeared to reduce DKD risk in diabetic patients. Additionally, they observed a higher prevalence of comorbidities, such as PAD, hyperlipidemia, coronary artery disease, and cerebrovascular events, among patients with DKD compared to those without DKD. These comorbidities were PAD (10.8% vs. 3.5%), hyperlipidemia, coronary artery disease, cerebrovascular accidents, congestive heart failure, carotid artery stenosis, aortic aneurysm, and osteoarthritis. However, in logistic regression, none of these were significant risk factors for DKD [28].

The relationship between PAD and diabetic nephropathy has been explored in several studies. For instance, Lee et al. demonstrated that macroalbuminuria was a stronger risk factor for PAD than reduced eGFR in patients with type 2 diabetes [29]. Similarly, Wattanakit et al. showed that albuminuria increases the risk of PAD by 1.9‐fold [30]. In line with our findings, Makhdoomi et al. reported a significant association between low ABI and microalbuminuria, even after adjusting for potential confounders [14]. Furthermore, studies have linked brachial‐ankle pulse wave velocity (baPWV)—a marker of arterial stiffness—with microvascular complications of diabetes, such as diabetic nephropathy and neuropathy [31, 32].

The mentioned association between PAD and DKD is explainable by shared pathophysiological mechanisms including atherosclerosis, inflammation, and oxidative stress [33]. Chronic inflammation is a hallmark of diabetes and is associated with endothelial dysfunction, which can lead to both microvascular (affecting the kidneys) and macrovascular (affecting peripheral arteries) complications [33]. However, our results showed that there is a significant relationship between these two conditions independent of shared risk factors.

Clear boundaries between the mechanisms underlying microvascular and macrovascular diabetic complications, and their respective responses to therapy, are difficult to draw [34]. Diabetic vasculopathy is increasingly viewed as a “panvascular” syndrome, in which macro‐ and microvessels across the cardiac, cerebral, renal, ophthalmic, and peripheral systems share common pathological features—principally endothelial dysfunction and atherosclerosis [35]. Shared mechanisms likely underlie these cross‐vascular associations, including AGE–RAGE signalling, oxidative stress, low‐grade inflammation, and downstream activation of NF‐κB, which together impair endothelial function and accelerate atherosclerotic plaque development and destabilisation [36, 37]. In a cohort study of 4083 patients with diabetes, microalbuminuria was associated with a 3.2‐fold higher risk of stroke incidence, while macroalbuminuria and end‐stage renal disease increased the risk by hazard ratios of 4.9 and 7.5, respectively [38]. These findings support the concept that microvascular complications may predispose individuals to macrovascular disease. Our study further aligns with evidence linking DKD to macrovascular complications in diabetes.

Several studies have pointed out that DKD is linked to vascular injury and an increased risk of cardiovascular events [39]. Increased aortic and arterial stiffness has been a suspect for mediating the link between DKD and cardiovascular disease. It has been shown that patients with CKD without diabetes also undergo vascular remodelling. Increased oxidative stress and AGEs production in diabetes can accelerate the endothelial injury and vascular stiffness [12]. Arterial stiffness correlates with lower ABI according to a study on 259 adults [13]. Another study showed that parameters of arterial stiffness such as total vascular resistance and augmentation index are higher in patients with PAD [40]. The association between PAD and DKD in our investigation could be explained by heightened arterial stiffness in DKD.

A recent concept suggests that macrovascular complications of diabetes may independently increase the risk of microvascular complications, beyond traditional shared risk factors. Zhang et al. demonstrated that PAD in patients with type 2 diabetes increased the relative risk of microvascular complications, including DKD, by 1.6‐fold [41]. Our findings support this concept, showing that PAD, as indicated by a low ABI, is independently associated with DKD, even after adjusting for conventional risk factors. Moreover, our results indicate that the severity of PAD is directly proportional to the risk of DKD. This underscores the importance of ABI as a reliable and easily measurable indicator for DKD risk assessment in routine clinical practice. Further longitudinal studies could clarify whether a low ABI is a risk factor for DKD or not.

### 4.1 Strengths and Limitations

This study adds to the limited body of research on the association of PAD and both DKD and albuminuria. Our findings confirm a significant association between macrovascular and microvascular complications, highlighting a relationship that extends beyond conventional shared risk factors. However, as with any other observational study, our investigation has some limitations. First, this was a cross‐sectional study and no causal relationship between PAD and DKD could be presumed based on our results. Second, this was a single‐center study which might limit its generalisability. Third, the use of oscillometric sphygmomanometers for ABI measurement, while practical, may not be as precise as methods such as Doppler ultrasound or toe‐brachial index assessments. Although Doppler ultrasound remains the gold standard for ABI measurement due to its superior accuracy and sensitivity, automated oscillometric devices have emerged as practical alternatives. Several studies have demonstrated moderate to good agreement between oscillometric and Doppler‐based ABI. A study on 286 patients compared the diagnostic accuracy of a semi‐automatic, four‐point oscillometric device with Doppler‐based ankle‐brachial index (dABI). ROC analysis showed that there were no significant differences between Oscillometric ABI and dABI in the detection of PAD [41]. Oscillometric devices offer benefits in terms of ease of operation, time efficiency, and operator independence, which makes them suitable for routine use in outpatient and research settings. In a study on 230 diabetic patients, the mean time for oscillometric ABI was 8.600 versus 16.980 min for Doppler ABI (*p* < 0.001) [18]. Fourth, we did not collect renal histopathological data, which could have provided deeper insights into the types of kidney injury associated with PAD. Future studies employing longitudinal designs and advanced diagnostic techniques will be essential to confirm and expand upon our findings. Although our findings revealed a statistically significant association between ABI ≤ 0.9 and DKD in the adjusted model (OR: 2.99, 95% CI: 1.05–8.4), the wide confidence interval suggests a degree of imprecision in the effect estimate. This variability may be attributed to the limited sample size and the relatively small number of patients in the lower ABI subgroup, which can affect the stability of the regression model. While the lower bound of the CI still indicates a clinically meaningful association, the upper bound implies a potentially stronger effect. This reinforces the need for larger, prospective studies to better quantify this relationship with greater precision and to confirm the consistency of our findings.

Another limitation of our study was that we recruited our patients from a tertiary centre that does not reflect the average population of type 2 diabetes patients. We also did not follow the patients due to the cross‐sectional design of the study. There were also other potential confounders that the models were not adjusted for, such as smoking and the type of medication.

## 5 Conclusion

This study demonstrates a significant association between PAD, assessed via ABI, and DKD in patients with type 2 diabetes. Our findings indicate that a reduced ABI (< 0.9) independently associates with both albuminuria and DKD, with greater odds observed at ABI values below 0.8. This underscores the utility of ABI as a practical, non‐invasive tool for identifying diabetic patients at high risk of DKD and guiding early interventions to mitigate disease progression. The high prevalence of PAD observed in this study emphasises the importance of the integration of ABI measurement into routine diabetes care to mitigate the progression of vascular complications in diabetes. Future longitudinal cohort and interventional studies are warranted to explore the causal relationship between PAD and DKD.

## Author Contributions

**Maryam Hedayati Moshkele:** writing – original draft, conceptualization, formal analysis, methodology. **Saeed Mirmoosavi:** writing – original draft, review and editing, formal analysis. **Mohammad Taghi Najafi:** writing – review and editing. **Sahar Karimpour:** writing – review and editing. **Alireza Esteghamati:** supervision, writing – review and editing. **Manouchehr Nakhjavani:** supervision, writing – review and editing. **Soghra Rabizadeh:** supervision, formal analysis, methodology, project administration, writing – review and editing.

## Conflicts of Interest

The authors declare no conflicts of interest.

## Data Availability

The data that support the findings of this study are available from the corresponding author upon reasonable request.
